# Supplementary figures and images for: Assessment of the effectiveness of public art in improving knowledge, attitude, practices and mitigation of stigmatization regarding neglected tropical diseases in South Eastern, Nigeria
Source: PLoS Negl Trop Dis. 2025 Jul 1;19(7):e0013266. doi: 10.1371/journal.pntd.0013266 (PMC12212551; doi:10.1371/journal.pntd.0013266)

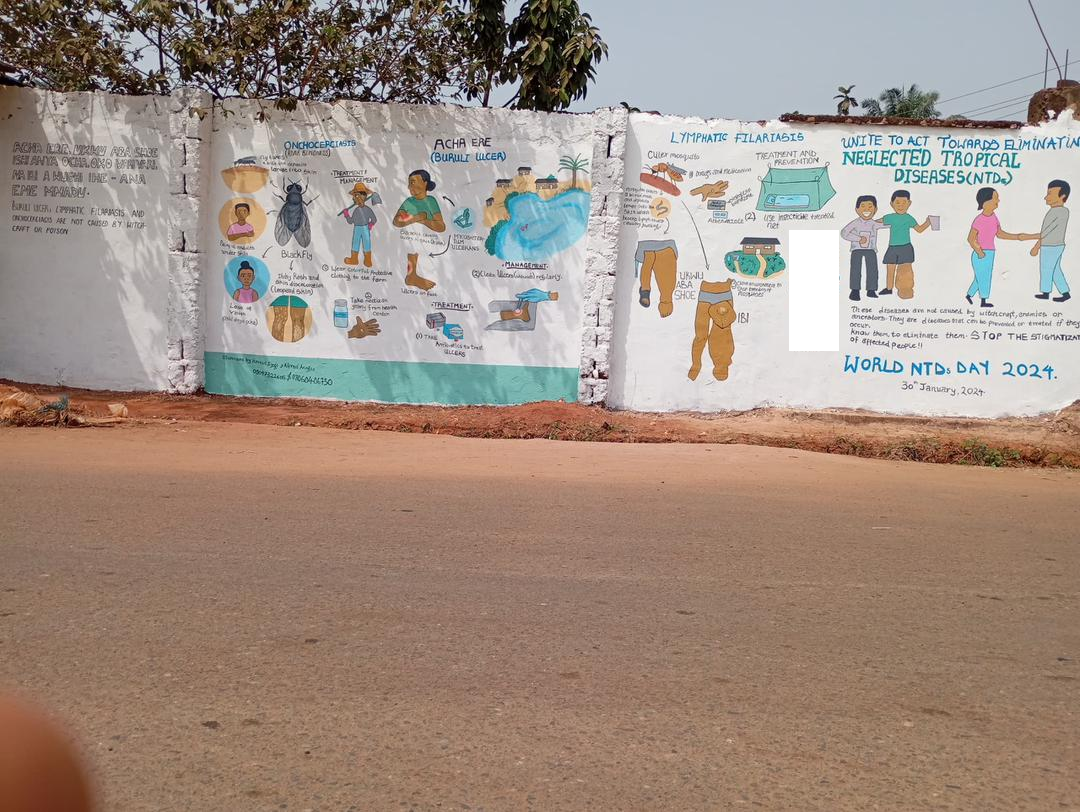

Supplement: S1 Public Art — (TIF) [file pntd.0013266.s001.tif]
