# Supplementary material for: Assessment of the effectiveness of public art in improving knowledge, attitude, practices and mitigation of stigmatization regarding neglected tropical diseases in South Eastern, Nigeria
Source: PLoS Negl Trop Dis. 2025 Jul 1;19(7):e0013266. doi: 10.1371/journal.pntd.0013266 (PMC12212551; doi:10.1371/journal.pntd.0013266)
Supplement: Attachment — Submitted filename: KAP on public Art Comments.pdf [file pntd.0013266.s003.pdf]

Public art as a health intervention represents an innovative approach for improving knowledge, attitudes, and practices regarding NTDs. The study brings to bear an approach that has not been really explored yet and so the study is a good one, however, the presentation and organization needs to be improved for publication:

Major comments:

The title ''**Using Community Public Art to Improve Knowledge, Attitude, Practices and Mitigation of Stigmatization Regarding Neglected Tropical Diseases in a Rural Endemic Community of South Eastern Nigeria''** seem too ambiguous and more than the conventional 25 words for title

I suggest

''Assessment of the Effectiveness of Public Art in improving Knowledge, Attitude, Practices and Mitigation of Stigmatization regarding Neglected Tropical Diseases in South Eastern, Nigeria''

The **method** section needs to be better organized: here is a simple guide that can be adapted and improved

- Research design
- Population and sample
- Research instrument
- Data collection instrument
- Data collection procedure
- Data analysis
- Ethical considerations

Line 128: this section should come later in the method section, not first like it currently appears and add ethical approval number

Line 139: Mixed method is a data collection method and not a research design, think experimental design is more appropriate for the study

There is also the need to clearly state what was done in the qualitative and quantitative methods separately ad clearly. Appears muddled up now

Describe each of the data collection instruments clearly

Line 144: authors can title the section: Advocacy visit then data collection procedure, which should come later

Provide inclusion and exclusion criteria under the methods

Line 155: How many GDS were carried out, across genders and age groups: usually young men, older men, young women, older women

Line 178: There is the need to add the info on the no. of villages in the community while describing the study area, there's a mention of 8 villages but it's not coming up in the sampling or anywhere else again… review

Line 187: Prevalence of which of the NTDs was used in sample size calculation, all 3 NTDS prevalent in the community or what, please add the info, not clear yet

Sampling technique: how many streets were there,

Line 186 What informed the choice of six households, provide justification

Was the same approach repeated for both post-test and pre-test

Line 192: explain clearly under the qualitative method of data collection

Line 197: Instrument(s), list questionnaire, FDG and KII guide separately

Line 209: subject expert instead of research expert

Line 210: Pilot test where?

Line 220: You may add intervention: Public Art design and display

Line 226: you have artist and the artists elsewhere line , how many were used

Line 232: you may add, produced and **displayed**

Line 234: **continuously** assessed the impact of the public art on community knowledge, attitudes, practices, and stigma through surveys and feedback, how… too vague, be more specific on what you did and for how long

Line 237: Post-intervention

Were the same set of people used at baseline surveyed or another group of people entirely?

How were they selected?

For the data analysis, separate analysis for quantitative and qualitative data and supply sub-headings

Lines 257-258 should come under the questionnaire description

Line 262: Paired sample t-tests were used to compare mean knowledge score, result of t-test not found

Result section: too wordy. Pls recast and summarize the tables

Line 299, all tables should no longer have questions but variables e.g, Have you heard of the diseases called Onchocerciasis, could be put as 'ever heard of onchocerciasis', apply to all tables,

Which group of individuals does NTDs mostly disproportionately affect can be put as "group mostly affected by NTDs

Line 366: You may sum up strongly agree and agree to have a single score, ditto for disagree and strongly disagree

Line 407: add 'had' experienced NTDs

Line 408: recast e.g. proportion prevented from staying in an assembly due to NTDs

References: the format for in-text citation is inconsistent, both numbers and names were used within text. Correct to journal format
