# Supplementary material for: Assessment of the effectiveness of public art in improving knowledge, attitude, practices and mitigation of stigmatization regarding neglected tropical diseases in South Eastern, Nigeria
Source: PLoS Negl Trop Dis. 2025 Jul 1;19(7):e0013266. doi: 10.1371/journal.pntd.0013266 (PMC12212551; doi:10.1371/journal.pntd.0013266)
Supplement: Attachment — Submitted filename: PA_Edits_PNTD-D-25-00200_reviewer.pdf [file pntd.0013266.s004.pdf]

PLOS Neglected Tropical Diseases

Using Community Public Art to Improve Knowledge, Attitude, Practices and Mitigation of Stigmatization Regarding Neglected Tropical Diseases in a Rural Endemic Community of South Eastern Nigeria

--Manuscript Draft--

Manuscript Number:	PNTD-D-25-00200
Full Title:	Using Community Public Art to Improve Knowledge, Attitude, Practices and Mitigation of Stigmatization Regarding Neglected Tropical Diseases in a Rural Endemic Community of South Eastern Nigeria
Short Title:	Community Public Art to Improve KAP and Stigma Mitigation Regarding NTDs
Article Type:	Research Article
Keywords:	Community Public Art, Neglected Tropical Diseases, Knowledge, Attitude and Practices
Abstract:	Background: Neglected Tropical Diseases (NTDs) continue to significantly impact marginalized communities, contributing to high morbidity, stigma, and social exclusion. Traditional health education strategies often fail to engage affected populations effectively. This study evaluates the impact of a community public art as an innovative tool for improving knowledge, shifting attitudes, enhancing preventive practices, and reducing stigma related to NTDs in Okwelle Community, Imo State, Nigeria.<br>Methodology/Principal Findings: A mixed-methods implementation research design was employed, involving 724 participants (362 at pretest and 362 at posttest). Public art installations depicting common NTDs in the study location including, Onchocerciasis, Buruli ulcer, and Lymphatic filariasis, were strategically placed in community spaces. Pretest and posttest surveys, key informant interviews, and focus group discussions were conducted to elucidate information about the influence of the public art on knowledge improvement and perception shifting. Quantitative data were analyzed using chi-square tests. Findings revealed a significant increase in NTD awareness post-intervention ($p < 0.05$). Identification of disease symptoms improved, misconceptions regarding supernatural causation decreased, and preventive behaviors such as healthcare-seeking and participation in community health programs increased. Stigma reduction was evident, with greater acceptance of affected individuals and increased willingness to interact with them. Notably, 98.3% of participants supported maintaining public art as an educational tool.<br>Conclusions/Significance: Public art can be a powerful and culturally relevant medium for enhancing NTD awareness, promoting behavior change, and reducing stigma in endemic communities. This study highlights its potential for integration into broader public health strategies to improve disease awareness and community participation. Future research should explore the scalability and long-term sustainability of public art interventions in diverse settings.<br>Author Summary<br>Neglected Tropical Diseases affect some of the world’s most vulnerable communities, causing illness, disability, and social stigma. Many people are unaware of these diseases or hold misconceptions that prevent early diagnosis and treatment. In this study, we explored how public art can be used as a tool to educate communities, improve knowledge, and reduce stigma associated with NTDs.<br>We worked with community members in Okwelle, Nigeria, to create public murals that visually represented common NTDs including, Onchocerciasis, Buruli ulcer, and Lymphatic filariasis. Before the artwork was introduced, many residents had limited knowledge of these diseases, and some believed they were caused by witchcraft or punishment from a higher power. After the murals were displayed, awareness increased significantly, attitudes toward affected individuals improved, and more people took steps to prevent and treat NTDs.<br>Our study shows that public art can be an effective and culturally relevant way to share health information, challenge stigma, and encourage healthier behaviors in underserved communities. This approach could be adapted for other public health challenges, making education more engaging and accessible to those who need it most.
Additional Information:	

Powered by Editorial Manager® and ProduXion Manager® from Aries Systems Corporation

Question	Response
**Government Employee**<br><br>Are you or any of the contributing authors an employee of the United States government?<br><br>Manuscripts authored by one or more US Government employees are not copyrighted, but are licensed under a CC0 Public Domain Dedication, which allows unlimited distribution and reuse of the article for any lawful purpose. This is a legal requirement for US Government employees.<br><br>This will be typeset if the manuscript is accepted for publication.	No - No authors are employees of the U.S. government.
**Financial Disclosure**<br><br>Enter a financial disclosure statement that describes the sources of funding for the work included in this submission. Review the submission guidelines for detailed requirements. View published research articles from *PLOS Neglected Tropical Diseases* for specific examples.<br><br>This statement is required for submission and **will appear in the published article** if the submission is accepted. Please make sure it is accurate.<br><br>**Funded studies**<br>Enter a statement with the following details:<br>• Initials of the authors who received each award<br>• Grant numbers awarded to each author<br>• The full name of each funder<br>• URL of each funder website<br>• Did the sponsors or funders play any role in the study design, data collection and analysis, decision to publish, or preparation of the manuscript?	The author(s) received no specific funding for this work.

Did you receive funding for this work?	
**Competing Interests**<br><br>On behalf of all authors, disclose any competing interests that could be perceived to bias this work.<br><br>This statement will be typeset if the manuscript is accepted for publication.<br><br>Review the instructions link below and PLOS NTDs' competing interests policy to determine what information must be disclosed at submission.	The authors have declared that no competing interests exist
**Data Availability**<br><br>Provide a **Data Availability Statement** in the box below. This statement should detail where the data used in this submission can be accessed. This statement will be typeset if the manuscript is accepted for publication.<br><br>Before publication, authors are required to make all data underlying their findings fully available, without restriction. Review our PLOS Data Policy page for detailed information on this policy. Instructions for writing your Data Availability statement can be accessed via the Instructions link below.	All relevant data are within the manuscript and its Supporting Information files

Powered by Editorial Manager® and ProduXion Manager® from Aries Systems Corporation

Manuscript
Click here to access/download;Manuscript;Community Public Art for NTDS_Manuscript.docx

Using Community Public Art to Improve Knowledge, Attitude, Practices and Mitigation of Stigmatization Regarding Neglected Tropical Diseases in a Rural Endemic Community of South Eastern Nigeria

Uchechukwu M. Chukwuocha
Department of Public Health, Federal University of Technology, Owerri, Nigeria

Ayoola O. Bosede
Department of Environmental Health Sciences, Federal University of Technology, Owerri, Nigeria

Chidubem D. Osuji
Department of Public Health, Federal University of Technology, Owerri, Nigeria

Adanna N. Chukwuocha
Department of Environmental Health Sciences, Federal University of Technology, Owerri, Nigeria

Harriet Abugewa
Department of Public Health, Federal University of Technology, Owerri, Nigeria

Amarachi Amawuru
Department of Public Health, Federal University of Technology, Owerri, Nigeria

Adesua Okoroh
Department of Public Health, Federal University of Technology, Owerri, Nigeria

Akuchi Echefula
Department of Public Health, Federal University of Technology, Owerri, Nigeria

Precious Nwabueze
Department of Public Health, Federal University of Technology, Owerri, Nigeria

Nathan Ukwenga
Department of Public Health, Federal University of Technology, Owerri, Nigeria

Alfred Barile
Department of Public Health, Federal University of Technology, Owerri, Nigeria

Abstract

Background: Neglected Tropical Diseases (NTDs) continue to significantly impact marginalized communities, contributing to high morbidity, stigma, and social exclusion. Traditional health education strategies often fail to engage affected populations effectively. This study evaluates the impact of a community public art as an innovative tool for improving knowledge, shifting attitudes, enhancing preventive practices, and reducing stigma related to NTDs in Okwelle Community, Imo State, Nigeria.

Methodology/Principal Findings: A mixed-methods implementation research design was employed, involving 724 participants (362 at pretest and 362 at posttest). Public art installations depicting common NTDs in the study location including, Onchocerciasis, Buruli ulcer, and Lymphatic filariasis, were strategically placed in community spaces. Pretest and posttest surveys, key informant interviews, and focus group discussions were conducted to elucidate information about the influence of the public art on knowledge improvement and perception shifting. Quantitative data were analyzed using chi-square tests. Findings revealed a significant increase in NTD awareness post-intervention ($p < 0.05$). Identification of disease symptoms improved, misconceptions regarding supernatural causation decreased, and preventive behaviors such as healthcare-seeking and participation in community health programs increased. Stigma reduction was evident, with greater acceptance of affected individuals and increased willingness to interact with them. Notably, 98.3% of participants supported maintaining public art as an educational tool.

Conclusions/Significance: Public art can be a powerful and culturally relevant medium for enhancing NTD awareness, promoting behavior change, and reducing stigma in endemic communities. This study highlights its potential for integration into broader public health strategies to improve disease awareness and community participation. Future research should explore the scalability and long-term sustainability of public art interventions in diverse settings.

Author Summary

Neglected Tropical Diseases affect some of the world's most vulnerable communities, causing illness, disability, and social stigma. Many people are unaware of these diseases or hold misconceptions that prevent early diagnosis and treatment. In this study, we explored how public art can be used as a tool to educate communities, improve knowledge, and reduce stigma associated with NTDs.

We worked with community members in Okwelle, Nigeria, to create public murals that visually represented common NTDs including, Onchocerciasis, Buruli ulcer, and Lymphatic filariasis. Before the artwork was introduced, many residents had limited knowledge of these diseases, and some believed they were caused by witchcraft or punishment from a higher power. After the murals were displayed, awareness increased significantly, attitudes toward affected individuals improved, and more people took steps to prevent and treat NTDs.

Our study shows that public art can be an effective and culturally relevant way to share health information, challenge stigma, and encourage healthier behaviors in underserved communities. This approach could be adapted for other public health challenges, making education more engaging and accessible to those who need it most.

Introduction

Neglected Tropical Diseases (NTDs) are a collection of chronic, debilitating infections that affect over one billion of the world's poorest individuals, primarily in rural areas with limited access to healthcare, clean water, and adequate sanitation. Concentrated in under-resourced regions across Africa, Asia, and Latin America, NTDs result in over 200,000 deaths annually and cause about 19 million disability-adjusted life years (DALYs), with estimates equating the collective burden of NTDs to that of HIV/AIDS, tuberculosis, and malaria combined (1). In rural communities, where economic opportunities are scarce, the disabling effects of NTDs trap affected individuals in a cycle of poverty, reducing productivity and increasing dependency (2,3). This creates an immense socioeconomic impact that hinders not only individual quality of life but also broader community development.

Beyond the physical and economic toll, NTDs carry a significant social burden. Individuals affected by NTDs often face stigmatization and discrimination due to the visible disfigurements and disabilities associated with these diseases (3). This stigma manifests as social exclusion, shame, and a reluctance to seek timely medical care. Conditions like lymphatic filariasis, leprosy, and Buruli ulcer, which cause noticeable physical signs, are frequently misunderstood by community members, leading to increased stigmatization and isolation (3). Such social ostracization exacerbates the challenges already faced by affected individuals, negatively impacting their mental well-being and further deepening health inequities. Moreover, stigma poses a major barrier to effective disease control efforts, as individuals may avoid diagnosis and treatment to escape judgment, inadvertently increasing disease transmission rates and impeding public health interventions (4).

To break the stigma surrounding NTDs, raising awareness and fostering positive perceptions within communities is crucial. Public health education initiatives can help demystify these diseases, addressing misconceptions and promoting empathy toward affected individuals(5,6). However, traditional awareness

campaigns often fail to engage rural populations effectively due to limited outreach, literacy barriers, and cultural disconnect (7). Community-centered approaches that incorporate locally relevant and accessible methods are needed to ensure effective knowledge dissemination and engagement. One such approach is the use of public art, a creative and culturally resonant medium that can capture public attention, invoke shared experiences, and promote collective understanding. Public art, created for and by the community, holds a unique capacity to engage audiences, communicate health messages, and influence public perceptions (8,9). Unlike conventional awareness materials, such as pamphlets or radio broadcasts, public art fosters interaction and dialogue, making health education a more immersive and participatory experience. By integrating key health messages into murals, sculptures, and other visual installations, public art can bridge communication gaps and encourage meaningful conversations about NTDs within affected communities. Furthermore, art can provide a sense of ownership and agency among community members, empowering them to take an active role in health advocacy and stigma reduction efforts.

This study explores the potential of community public art under the initiative "Public Art for Public Health" as an innovative tool for promoting NTD awareness, enhancing knowledge, and reducing stigmatization in a rural community in Imo State, Nigeria. By assessing community knowledge, perceptions, and stigma levels both before and after the deployment of awareness-raising artwork, this research aims to demonstrate the effectiveness of public art in shifting public attitudes and fostering social inclusion. The study further seeks to highlight how art-driven interventions can complement existing health programs, contributing to broader NTD control efforts by addressing not only the medical but also the social determinants of these diseases.

Material and Methods

Ethics statement

This study received ethical approval from the Department of Public Health, Federal University of Technology, Owerri, Nigeria's research ethics committee before data collection began. All participants were fully informed about the purpose, procedures, and potential benefits of the study, and written informed consent was obtained from each participant prior to their involvement. Participation in the study was voluntary, and participants had the right to withdraw at any stage without any consequence. To ensure privacy and confidentiality, all personal identifiers were removed and data collection instrument anonymized. Data were securely stored and accessible only to the research team. Results were reported in aggregate form, with no individual identifiers disclosed, thereby safeguarding participant privacy. These measures adhered to ethical standards for research involving human participants, ensuring the protection of participants' rights and well-being throughout the study.

Study design

This study utilized a mixed-methods approach within an implementation research framework to evaluate the impact of public art interventions on knowledge, perceptions, and stigma related to NTDs in Okwelle Community, Onuimo Local Government Area, Imo State. The study was part of the Public Art for Public Health initiative, where a team of researchers used strategically placed public artwork to foster knowledge improvement, shift perceptions, and mitigate stigma associated with NTDs.

Community engagement, stakeholder identification, and Public Art deployment

A crucial first step involved engaging with community leadership in a focus group discussion (FGD) to assess the suitability of the already sketched artwork within the local context and to secure a highly accessible display location for all community members. Formal inquiries were made at the community

health center through the chief nurse to identify key stakeholders who influence health opinions in the community. These stakeholders included the Traditional Ruler, Town Union Chairman, Traditional Herbalists, Market Union Leaders, Youth Leader, Women Leader, health workers, and Religious Leaders. A purposive sampling strategy was employed to achieve variation across these stakeholder groups while ensuring gender, age, and role diversity. To mitigate research bias inherent in non-probabilistic sampling, only stakeholders who had resided in the community for at least seven years and demonstrated an understanding of the local health dynamics were selected. Twelve (12) designated stakeholders participated in the FGDs that led to the final sketch of the artwork and the identification of the location where the artwork was painted.

During the engagement sessions, stakeholders were introduced to the concept of using public art as a medium for NTD awareness, specifically focusing on Buruli ulcer, onchocerciasis, and lymphatic filariasis. Discussions were conducted in the Igbo language to ensure full comprehension among participants. The team explained that NTDs are not hereditary or supernatural ailments but are linked to environmental conditions and pathogenic factors, translated in Igbo as: *'Oria ndia abughi ihe na-efe efe, kama, o bu ihe n'esita na nje, nke n'esita na obibi anyi na usoro ime ihe n'ulo anyi di iche iche'.*

Stakeholders actively engaged in discussions, asked clarifying questions, and provided insights. They contributed suggestions to refine the concept before finalizing and displaying the artwork. Their involvement extended to identifying a suitable location for the artwork within the community to ensure maximum visibility and engagement.

Study area

The study was carried out in the Okwelle community, Onuimo LGA, Imo State, Nigeria. This community has a population of approximately 9,247 (2024 projection of 2006 census) and is situated at latitude 5°45'5''N and longitude 7°10'39''E, with a landmass of approximately 8.5 km². Known for its central

market, Okwelle attracts traders from surrounding towns in southeastern Nigeria, and the local economy is primarily based on agriculture, trade, and civil service employment. The community has only one Basic Health Center that attend mostly to maternal and child health cases. A number of Lymphatic filariasis, Buruli ulcer and Onchocerciasis cases were sighted during data collection, confirming the prevalence of NTDs in the community.

Study population

The study population included all adults (18 years and above) residing within the Okwelle community for at least one year prior to the study. Participants were selected from each of the eight villages to ensure comprehensive representation.

Sample size

The sample size for quantitative data collection was calculated using the Leslie Kish formula (10), where the estimated proportion of the target population (p) was based on similar studies (11). With a prevalence estimate (p) of 63.1% from prior studies, the minimum sample size was calculated as 357.6, rounded to 361 to account for a 10% non-response rate.

Sampling technique

For the quantitative survey, the community was stratified into streets, each assigned a unique number for easy identification. Systematic random sampling was then employed to select households. The first household on each street was chosen randomly, followed by every sixth household. If a selected household lacked a suitable respondent or if the respondent was not interested in participating, the next eligible household was selected, while maintaining the interval of every sixth household until the required sample size was reached.

For the Key Informant Interviews (KIIs), conducted three months after the deployment of the public art as part of the follow-up study, a purposive sampling approach was used in collaboration with the basic health center. Key informants were identified and recruited based on their knowledge and influence on health-related matters within the community. Interviews continued until data saturation was achieved, resulting in a total of 16 key informants participating in the study.

Instrument for Data Collection

Data were collected using a structured questionnaire administered through the Open Data Kit (ODK) tool, which enabled efficient data gathering in resource-constrained settings. The questionnaire included sections on demographic characteristics, knowledge and perceptions of NTDs, and attitudes toward individuals with NTDs. Closed-ended questions with multiple-choice options and Likert scale items were used to capture respondents' attitudes and perceptions.

A semi-structured key informant interview guide was used to collect qualitative data from key informants. The guide included prompts that encouraged informants to freely discuss their perceived changes in knowledge, attitudes, and stigma associated with NTDs within the community. Additionally, informants provided insights on the suitability and placement of the public art, as well as their observations of community engagement and interaction with the artwork.

Validity and reliability of instrument

To ensure validity, the questionnaire was reviewed by a research expert and the study supervisor, focusing on face and content validity. A pilot test of the instrument was conducted, and the reliability of the questionnaire was assessed using Cronbach’s alpha, yielding a reliability coefficient of 0.78, which meets the acceptable threshold for high reliability.

Baseline data collection

Baseline data collection was done to establish initial information on the community's knowledge, perceptions, and stigmatization regarding NTDs. Data gathered included participants' awareness of NTD transmission, prevention, and treatment options. The baseline data served to identify gaps in knowledge and common stigmatizing attitudes, informing the design and objectives of the subsequent public art intervention.

Public Art intervention design and implementation

A local Artist was engaged to design and create public art installations that conveyed information about NTDs, their prevention, and available treatments. A search was conducted to identify talented and respected local Artists, followed by meetings to discuss the project and gauge their interest. A community workshop was held to introduce the project to both artists and residents, generating interest and support by highlighting the role of art in raising awareness about NTDs.

The selected Artist collaborated with researchers to conceptualize the installations, ensuring key messages and visuals effectively conveyed NTD-related information. Inspiration was drawn from local culture, traditions, and community values. Artists were provided with relevant NTD data and educational materials, while healthcare professionals reviewed the content for medical accuracy.

Once the design was finalized, Artists created murals, allowing community members to observe, ask questions, and engage in discussions. Interactive workshops were held involving residents in painting and decorating, fostering a sense of ownership and involvement. The murals were produced in prominent public spaces for easy access by everyone in and around the community (Plate 1).

The study continuously assessed the impact of the public art on community knowledge, attitudes, practices, and stigma through surveys and feedback. Ongoing collaboration with artists and the community ensured the artwork remained a valuable educational resource for NTD awareness.

Follow-up data collection

A follow-up data collection was conducted three months after the installation of the public art to evaluate its influence on community knowledge, perceptions, and stigmatization regarding NTDs. This follow-up aimed to assess changes in the target population's understanding of NTD transmission, prevention, and treatment, as well as shifts in attitudes toward individuals affected by NTDs. The follow-up survey used the same questionnaire as the baseline data collection to ensure consistency in measuring changes. Data on knowledge scores, perceptions, and stigmatization levels were collected and compared with baseline data to assess the effectiveness of the public art intervention.

Data analysis

This study collected two sets of data. The first set comprised baseline data collected before the deployment of the public artwork, aimed at assessing community knowledge about NTDs—including modes of transmission, prevention strategies, treatment options, and management. The baseline data also gauged perceptions of NTDs and the level of associated stigma. Three months after the artwork installation, a follow-up data collection was conducted using the same instrument with minor additions. This second phase gathered information on how many respondents had engaged with the artwork, their opinions of it, and whether they wanted it to remain in the community.

All collected data were initially processed in Microsoft Excel for cleaning, sorting, and coding, then imported into SPSS (12) for analysis. In SPSS, demographic information was analyzed using descriptive statistics and presented as frequency distributions and percentages, with certain variables displayed in tables and charts.

The knowledge assessment tool included several questions related to NTDs such as Onchocerciasis, Buruli ulcer, and Lymphatic filariasis. The answers to these questions were presented as frequency of correct responses, there frequency were compared with the posttest responses and Chi-square test was used to test the statistical significance of the responses for the pre- and the posttest groups. Categorical variables were presented as frequencies and percentages, and continuous variables were summarized with means and standard deviations. Paired sample t-tests were used to compare mean knowledge scores before and after the public art intervention.

Perceptions of NTDs were analyzed using a Likert scale, with baseline and follow-up frequencies displayed side-by-side. Stigmatization was assessed in two ways;

1. Respondents with NTDs or relatives with NTDs were asked if they had experienced stigma, with responses presented in a frequency distribution table, comparing baseline and follow-up responses.

2. Respondents without NTDs were asked if they would stigmatize others with NTDs. Their responses were also presented side-by-side with baseline and follow-up data to assess any changes in attitudes following the intervention.

The transcripts from the KIIs captured all spoken words, including non-verbal cues and pauses, ensuring a comprehensive record of the discussions. Each interview was stored as separate documents for systematic organization. NVivo, was employed to efficiently manage, code, and analyze the data. A coding framework was developed to tag and classify key themes, concepts, and variables relevant to the research. This structured approach facilitated the identification of patterns and insights, enabling a deeper understanding of community perspectives on NTDs, public art engagement, and stigma reduction.

Results

Sociodemographic Characteristics of the Study Group

There were two groups of 362 persons each studied at pretest and posttest. The sociodemographic characteristics of the study groups are presented in table 4.1., and shows that the characteristics of both groups were quite similar. The average age of the participants was 45.1 years in the pretest, at a corresponding standard deviation (SD) of 16.5 years. For the posttest group, the mean age was 44.8 years (SD=12.3 years). Close to half at each group had secondary education level (pretest = 49.9%, posttest= 48.9%), while those that had tertiary education level were 107 (30.1%) for pretest group and 123 (34%) for posttest group. In terms of marital status, majority were married in both groups (pretest =66.2%; posttest = 70.7%). The occupational status for the pretest group includes 47.3% self-employed, 27% employed and 25.6% unemployed. For the posttest we have self-employed (45%), employed (34.5%) and unemployed (20.4%). A total of 31.3% and 31.2% respectively in pretest and posttest were doing hand work, while 29.9% were involve in trading in pretest group (posttest =29%).

The less than thirty thousand naira (N30,000) monthly income earners make up 30.4% of the pretest group (11.3% in posttest group), while about 4.5 % in the pretest earn at least a N120,000 in a month (20.2% in posttest). Those with household size of 1-3 persons in the pretest group were 148 (41.7%), while 191 (52.8%) of the same household size were in the posttest group.

Table 1: Sociodemographic Characteristics of the Study Group

Sociodemographic Factors	Pretest		Post test	
	**Freq**	**Percent (%)**	**Freq**	**Percent (%)**
**Age**	Mean = 45.10	SD= 16.47	Mean = 44.81	SD= 12.29
**Educational attainment**				
Secondary school	177	49.86	177	48.9
Tertiary	107	30.14	123	33.98
Primary school	52	14.65	39	10.77
Others	19	5.35	23	6.35
**Marital Status**				
Married	235	66.2	256	70.72

Single	76	21.41	46	12.71
Widowed	33	9.3	30	8.29
Divorced	11	3.1	30	8.29
**Occupational status**				
Self Employed	168	47.32	163	45.03
Employed	96	27.04	125	34.53
Unemployed	91	25.63	74	20.44
Occupation				
Hand work	111	31.27	113	31.22
Trading	106	29.86	105	29.01
Farming	72	20.28	63	17.4
White **collar**	66	18.59	81	22.38
**Income level**				
N0- N30,000	108	30.42	41	11.33
N31,000-N60,000	103	29.01	115	31.77
N61,000-N90,000	81	22.82	49	13.54
N90,000-N120,000	47	13.24	84	23.2
N120,000 and above	16	4.51	73	20.17
**Number of people living in household**				
1-3persons	148	41.69	191	52.76
4-6persons	158	44.51	156	43.09
7 persons and above	49	13.8	15	4.14

Knowledge of Onchocerciasis at Pretest and Posttest

Table 2 shows that awareness of Onchocerciasis increased from 28.5% (103 respondents) at pretest to 39% (141 respondents) at posttest, with a significant difference ($p = 0.036$, $\chi^2 = 4.409$). Identification of Onchocerciasis manifestations also improved significantly from 41.7% to 53.0% ($p = 0.002$, $\chi^2 = 9.313$).

Understanding that NTDs can affect everyone rose significantly from 25.1% at pretest to 40.9% at posttest, while uncertainty decreased from 71% to 51.7% ($p = 0.000$, $\chi^2 = 29.44$). Awareness of associated problems increased, with disability (18% to 33%), loss of employment (19% to 23%), poor health (13.3% to 19.6%), and death (5.8% to 14.9%) all showing significant improvement. The proportion of respondents unaware of NTD-related problems declined from 40.3% to 8.8% ($p = 0.000$, $\chi^2 = 184.74$).

Knowledge of Onchocerciasis transmission via black fly bites increased from 40.1% (140 respondents) to 54.7% (198 respondents). Understanding that Onchocerciasis is not transmitted person-to-person

improved from 28.2% to 54.4%. Awareness of preventive measures increased, with responses for vaccination decreasing from 33.1% to 20.4% and avoiding black fly bites from 24.9% to 49.7%. Adoption of protective measures, including personal hygiene, wearing long sleeves, and using insect repellents, also improved.

At pretest, 49.4% (179 respondents) were unaware of treatment options for Onchocerciasis, but posttest findings showed that 61.9% recognized Ivermectin/Mectizan as a treatment. Significant improvements were observed across all knowledge areas, except for prevention methods, which showed borderline significance (p = 0.069).

Table 2: Knowledge of Onchocerciasis at Pretest and Posttest

Knowledge of Onchocerciasis	PRETEST		POSTTEST		Chi-sq	
	Freq	%	Freq	%	(χ^2)	P
**Have you heard of the diseases called Onchocerciasis**						
Yes	143	39.5	171	47.2		
No	219	60.5	191	52.8	4.409	0.036
**Identify Onchocerciasis manifestation from the photo album**						
Correct	151	41.7	192	53.0		
Incorrect	211	58.3	170	47.0	9.313	0.002
**Which group of individuals does NTDs mostly disproportionately affect**						
It affects everyone equally	91	25.1	148	40.9		
Men	9	2.5	14	3.9		
Women	4	1.1	9	2.5		
Children	1	0.3	4	1.1		
Don't know	257	71.0	187	51.7	29.440	0.000
**Mention some problems that are associated with NTDs**						
Disability	65	18.0	120	33.1		
Loss of employment	69	19.1	83	22.9		
Poor health	48	13.3	71	19.6		
Death	21	5.8	54	14.9		
Spending on healthcare and economic impoverishment etc	13	3.6	32	8.8		

Don’t know	146	40.3	2	0.6	184.74	0.000
**How do you think people get Onchocerciasis?**						
Poor hygiene	65	18.0	89	24.6		
Infection through bites of black flies	145	40.1	198	54.7		
Witchcraft	60	16.6	22	6.1		
Affliction by the gods	32	8.8	9	2.5		
Unknown	60	16.6	44	12.2	44.91	0.000
**Can Onchocerciasis transmit from person to person in the community?**						
I don't know	186	51.4	133	36.7		
No	102	28.2	197	54.4		
Yes	74	20.4	32	8.8	55.63	0.000
**How can Onchocerciasis be prevented?**						
I Don’t know	102	28.2	78	21.5		
Vaccination	120	33.1	74	20.4		
Avoiding being biting by blackflies	90	24.9	180	49.7	6.758	0.030
Avoiding being biting by blackflies						
Appeasing the gods through sacrifice	20	5.5	20	5.5		
Cannot be prevented	21	5.8	7	1.9		
Spiritual fortification	9	2.5	3	0.8	5.333	0.069
**What is the personal protection measures to prevent Onchocerciasis?**						
Personal hygiene	72	19.9	98	27.1		
Wearing of long sleeves and trousers	66	18.2	86	23.8		
Wearing of insect repellant	58	16.0	85	23.5		
Wearing of permethrin-treated clothes	52	14.4	39	10.8		
Eating good food	54	14.9	10	2.8		
Community spiritual cleansing	33	9.1	23	6.4		
Appeasing the gods	21	5.8	12	3.3		
Going to bed early	6	1.7	9	2.5	48.65	0.000
**What are the treatments choices of onchocerciasis?**						
Don’t know	179	49.4	109	30.1		
Ivermectin/Mectizan	157	43.4	224	61.9		
Rifampicin	19	5.2	21	5.8		
Doxycycline	7	1.9	8	2.2	28.96	0.000

Knowledge of Buruli Ulcer at Pretest and Posttest

Table 3 shows that awareness of Buruli ulcer increased from 53.6% (193 respondents) at pretest to 75.7% (274 respondents) at posttest. Identification of Buruli ulcer manifestations from a photo album improved from 52.2% to 71.5% posttest.

Misconceptions about the disease also reduced. At pretest, 49% believed Buruli ulcer was caused by poison, while at posttest, 48.6% correctly identified bacteria as the cause. Knowledge that Buruli ulcer is not transmitted person-to-person increased significantly from 28.2% to 61.9% posttest.

Awareness of personal protective measures improved, with 40.6% at posttest acknowledging the use of insect repellents on exposed skin compared to 19.3% at pretest. Similarly, 30.1% recognized the importance of wearing gloves, long sleeves, and trousers, up from 11.3% at pretest.

Regarding treatment knowledge, 74% (268 respondents) at pretest were unaware of the availability of safe and effective medication for Buruli ulcer, which significantly decreased to 25.4% at posttest.

Table 3: Knowledge of Buruli Ulcer at Pretest and Posttest

Knowledge of Neglected Tropical Diseases	PRETEST		POSTTEST			
	Freq	%	Freq	%	(χ^2)	
**Knowledge of Buruli Ulcer**						
**Have you heard about Buruli ulcer?**						
Yes	194	53.6	274	75.7		
No	168	46.4	88	24.3	40.186	0.000
**Identify Buruli ulcer manifestation from the photo album?**						
Correct	189	52.2	259	71.5		
Incorrect	5	1.4	22	6.1	5.905	0.015
**What is/are the causes of Buruli ulcer?**						
Poison	178	49.2	43	11.9		
Witch craft	126	34.8	35	9.7		
Bacteria	97	26.8	176	48.6		
Curse	58	16.0	11	3.0		
Insect bites	21	5.8	108	29.8	237.77	0.000

**Can Buruli ulcer be transmitted from one person to the other?**						
No	102	28.2	224	61.9		
Don't know	191	52.8	99	27.3		
Yes	69	19.1	39	10.8	83.18	0.000
**What are the personal measures to be taken to prevent Buruli ulcer?**						
Promptly wash scratches or cuts with soap	129	35.6	59	16.3		
Use of topical antiseptic and dressing on wound	116	32.0	64	17.7		
Wearing insect repellent on any exposed skin	122	33.7	206	56.9		
BCG vaccination	61	16.9	71	19.6		
Wearing of gloves, long sleeved and trousers	41	11.3	109	30.1	92.70	0.000
**What is/are the safe and effective medication available for the treatment of Buruli ulcer?**						
Don’t know	268	74.0	92	25.4		
Rifampicin	36	9.9	109	30.1		
Streptomycin	44	12.2	106	29.3		
Clarithromycin	14	3.9	96	26.5	207.95	0.000

Knowledge of Lymphatic Filariasis at Pretest and Posttest

Table 4 indicates that awareness of lymphatic filariasis increased from 57.7% (209 respondents) at pretest to 63.5% (230 respondents) at posttest. Identification of lymphatic filariasis manifestations from a photo album improved significantly from 33.1% (120 respondents) at pretest to 61.3% (222 respondents) at posttest.

Understanding of disease causation also improved. At pretest, only 15.5% and 10.8% correctly identified mosquito bites and a dirty environment as causes, which increased to 37% and 29.8%, respectively, at posttest. The proportion of respondents unaware of the transmission mode dropped from 41.2% to 26.8% posttest.

Knowledge of preventive measures also showed substantial improvement. At posttest, 37.0% identified wearing protective clothing, up from 16.3% at pretest, while recognition of mosquito bite avoidance as a preventive measure increased from 22.1% to 35.4%.

More than half (53%) of respondents were unaware of safe and effective medication for lymphatic filariasis at pretest, but this decreased significantly to 23.8% at posttest. At a 5% significance level, all posttest changes were highly significant (p = 0.000), except for general awareness of lymphatic filariasis.

Table 4: Knowledge of Lymphatic Filariasis at Pretest and Posttest

**Knowledge of Lymphatic filariasis**	**PRETEST**		**POSTTEST**			
	**Freq**	**%**	**Freq**	**%**	(χ^2)	P
**Have you heard about Lymphatic filariasis?**						
Yes	209	57.7	230	63.5		
No	153	42.3	132	36.5	2.552	0.110
**Identify Lymphatic filariasis manifestation from the photo album?**						
Correct	120	33.1	222	61.3		
Incorrect	242	66.9	140	38.7	57.657	0.000
**What are is/are the causes of Lymphatic filariasis?**						
Don't know	105	29.0	69	19.1		
Witcraft	74	20.4	17	4.7		
Affliction	63	17.4	15	4.1		
Mosquito bite	56	15.5	134	37.0		
Dirty environment	39	10.8	108	29.8		
Eating contaminated food	23	6.4	19	5.2		
Sexual intercourse	2	0.6	0	0.0	139.48	0.000
**What is the mode of transmission of Lymphatic filariasis**						
Don't know	149	41.2	97	26.8		
Affliction	94	26.0	46	12.7		
Mosquito bites	90	24.9	176	48.6		
Sharing contaminated clothing	29	8.0	43	11.9	57.98	0.000
**Mention how Lymphatic filariasis can be prevented**						
I don't know	149	41.2	77	21.3		

Wearing protective clothing	59	16.3	134	37.0		
Avoid mosquito bites	80	22.1	128	35.4		
Appeasing the gods	39	10.8	12	3.3		
Spiritual sacrifice	35	9.7	11	3.0	89.98	0.000
**What is/are the safe and effective medication available for the treatment of lymphatic filariasis?**						
Don’t know	192	53.0	86	23.8		
Albendazole/Ivermectin	59	16.3	98	27.1		
Ivermectin alone	47	13.0	90	24.9		
Diethylcarbamazine citrate	21	5.8	23	6.4		
Albendazole alone	11	3.0	23	6.4	67.80	0.000

The improvement in knowledge and awareness is also shown in the KII where the community leaders expressed good awareness and knowledge of the NTDs due to the display of public art in the community.

‘’I can vividly see what the NTDs are all about from the picture display’’ (TUC).

*‘’I can now understand that the display about NTDs is not against herbal remedies to cure of the diseases. (TH3)***.**

*‘’We always talk about the public art in the market and my members can now understand that these are curable and preventable disease’’ (MUL1)***.**

“I’m glad that this public art has made many to understand that NTDs are real” (CHW1)

Attitudes towards NTDs in the study area

Table 5 indicates improved attitude towards NTDat posttest, as many disagreed and strongly disagreed against wrong insinuations on the NTDs. At pretest, over 65% disagreed and strongly disagreed (disagreed: 34.3%, strongly disagreed: 30.9%) that NTDs are agelong diseases that can be inherited from generation to generation. This increased at posttest to 83.1% (disagreed: 38.1% and strongly disagreed: 45%). Similarly, many also disagreed that “NTDs are repercussion for sinful act against God/Allah” at posttest (36.2% to 49.4%) and about three folds strongly disagreed at posttest that Most NTDs are infectious diseases which are contracted when people come in contact with the infectious agents (15.5% to 46.4%). Other items also showed reasonable improvement in opinions and attitude against NTDs from pretest to

posttest. These include NTDs is caused as a result of manipulation from witchcraft (strongly disagreed: 23.5% to 43.5%), NTDs only affect the poor (strongly disagreed: 30.9% to 45%) and NTDs cannot be prevented, it comes whenever it wants to come (strongly disagreed: 30.7% to 44.6%).

Table 5: Attitude towards NTDs in the study Area

Attitude	PRETEST		POSTTEST	
	Freq	%	Freq	%
**NTDs are agelong diseases that can be inherited from generation to generation**				
Strongly disagree	122	30.9	163	45.0
Disagree	114	34.3	138	38.1
Agree	92	25.4	42	11.6
Strongly agree	34	9.4	21	5.8
**NTDs are repercussion for sinful act against God/Allah**				
Strongly Disagree	131	36.2	179	49.4
Disagree	104	28.7	109	30.1
Agree	92	25.4	48	13.3
Strongly agree	35	9.7	26	7.2
**Most NTDs are infectious diseases which are contracted when people come in contact with the infectious agents**				
Strongly disagree	56	15.5	168	46.4
Disagree	116	32.0	108	29.8
Agree	141	39.0	62	17.1
Strongly agree	49	13.5	24	6.6
**NTDs is caused as a result of manipulation from witchcraft**				
Strongly disagree	85	23.5	164	45.3
Disagree	75	20.7	106	29.3
Agree	163	45.0	72	19.9
Strongly agree	39	10.8	20	5.5
**There is no treatment for NTDs**				
Strongly Disagree	102	28.2	173	47.8
Disagree	120	33.1	120	33.1
Agree	103	28.5	44	12.2
Strongly agree	37	10.2	25	6.9
**NTDs only affects the poor**				
Strongly disagree	112	30.9	163	45.0
Disagree	120	33.1	127	35.1
Agree	105	29.0	53	14.6
Strongly agree	25	6.9	19	5.2
**NTDs cannot be prevented, it comes whenever it wants to come**				
Strongly Disagree	111	30.7	165	44.6

Disagree	120	33.1	119	32.9
Agree	102	28.2	69	19.1
Strongly agree	29	8.0	19	5.2

Effect of Public Art in improving the practices of NTDs control

Those who sought medical treatment or advice for any NTD-related symptoms or conditions for themselves or a family member in the past year increased from 26% at baseline to 59.1% at post intervention. Frequent engagement in practices that can help prevent NTDs, such as personal hygiene, sanitation, or use of protective measures increased from 25.1% to 46.4% (from baseline to post intervention), while those that never engaged in such also reduced from 8.6% to 4.4%.

Just 47.8% respondents participated in community health programs or initiatives related to NTDs in the area prior to this study but that rose up to 71.3% at post intervention.

Also, the extent to which people actively shared information about NTDs and their prevention methods with friends and family improved at post intervention (from 37.8% to 68.0%).

Those who believed that drugs for treatment of NTDs should be prescribed by a doctor before taking them were high at baseline (73.5%), but increased further at postintervention (85.4%). Statistical test at 5% level shows that the introduction of the public art has significant effects in improving the control practices of NTDs among community members in the study area ($p < 0.05$).

Table 6: Effect of Public Art in improving the control practices for NTDs among community members in study area

	PRETEST		POSTTEST			
**Practices for NTDs Control**	**Freq**	**%**	**Freq**	**%**	**(χ^2)**	**P**
**Have you personally sought medical treatment or advice for any NTD-related symptoms or conditions for yourself or a family member in the past year?**						
Yes	94	26	214	59.1		
No	268	74	148	40.9		
Total	362	100	362	100	8.220	0.004

**How often do you engage in practices that can help prevent NTDs, such as personal hygiene, sanitation, or use of protective measures?**						
Frequently	91	25.1	168	46.4		
Occasionally	157	43.4	121	33.4		
Rarely	82	22.7	57	15.7		
Never	31	8.6	16	4.4		
Total	362	100	362	100	36.836	0.000
**Are there any community health programs or initiatives related to NTDs in your area that you have participated in?**						
Yes	173	47.8	258	71.3		
No	189	52.2	104	28.7		
Total	362	100	362	100	41.422	0.000
**In your daily life, do you actively share information about NTDs and their prevention with friends and family?**						
Yes	137	37.8	246	68.0		
No	225	62.2	116	32.0		
Total	362	100	362	100	65.86	0.000
**The treatment drugs for NTDs should be prescribed by a doctor before taking them**						
TRUE	266	73.5	303	85.4		
FALSE	96	26.5	52	14.6		
Total	362	100	362	100	22.73	0.000

The responses for practices against NTDs were generally encouraging after the public art and education to the community about NTDs. Here are some of the responses from the KII that buttress this assertion

“This art work is highly welcomed in our society and we expect it to guide our community against NTDs” (TR)

“Many are now attending hospitals for screening for NTDs which is good news” (CHW1)

“*Women have been very motivated by this work and we have been mobilizing people in that condition for hospital treatment”* (WL2)

“Many of our youths have participated in training and workshops on NTDs recently and our youth association will continue to encourage active participations to clear out these diseases” (YL1).

Effect of public art in reducing social stigmatization surrounding NTDs

Table 7 shows that those that experience of NTDs did not differ much at pretest and posttest, but the proportion prevented from an assembly due to NTDs were slightly reduced at posttest (14.9% to 11.6%). Among this group, those prevented from an assembly due to NTDs were 55.6% and 40.5% respectively at pretest and posttest, and those denied entrance into a place because of the disease were 52.6% at pretest and 46.6% at posttest. Similar results were obtained on having handshake, having difficulties, employment denials, being isolated and others as a result of NTDs.

While only 27.9% are willing to sit in the same car with anyone with any NTD during pretest, the proportion increased to 42% at posttest. Those who responded that they would allow their children to attend school where there is a known NTD case were 43.1% at posttest against 22.5% at pretest. At posttest, the willingness to employ anyone with NTDs improved to 32.3% at posttest as against 14.9% at pretest, while 43.1% indicated that they would continue to keep NTDs persons as friends versus 30.4% at pretest.

Significant effects against stigmatization were observed including in the following areas; readiness to sit in the same car with anyone with any NTDs (p =0.000, χ^2=22.07), readiness to allow their children to attend school where there is a known NTD case (p =0.000, χ^2=45.42), readiness to employ anyone with NTDs (p =0.000, χ^2=36.20), agreed to continue keeping a friend who may suddenly develop NTDs (p =0.000, χ^2=18.14), ready to you patronize or buy items from a seller with any of the NTDs (p =0.000, χ^2=21.97) and being ready to render assistance to anyone with obvious NTD (p =0.000, χ^2=12.044).

Table 7: Effect of public art in reducing social stigmatization in the study area

	PRETEST		POSTTEST			
**Likely Stigmatization factors**	**Freq**	**%**	**Freq**	**%**	**(χ^2)**	**P**
**Have you or anyone around you experience any of the NTDs?**						
Yes	95	26.8	103	28.5		
No	260	73.2	259	71.6	0.257	0.612
**If yes, have you or any person you know been prevented from an assembly due to**						

**NTDs? (n=95 at pretest, 103 at posttest)**						
Yes	53	55.6	42	40.8		
No	42	44.4	61	59.2	4.463	0.035
**Have you or any person you know been denied entrance into a place because of NTDs? (n=95 at pretest, 103 at posttest)**						
Yes	50	52.6	48	46.6		
No	45	47.4	55	53.4	0.719	0.397
**Have you or any person you know been denied a handshake due to NTDs? (n=95 at pretest, 103 at posttest)**						
No	52	54.7	50	48.5		
Yes	43	45.3	53	51.5	0.759	0.384
**Have you or any person you know had/have difficulties with relationships due to NTDs? (n=95 at pretest, 103 at posttest)**						
Yes	51	53.7	45	43.7		
No	44	46.3	58	56.3	1.977	0.160
**Have you or any person you know been denied employment due NTDs? (n=95 at pretest, 103 at posttest)**						
Yes	51	53.7	48	46.6		
No	44	46.3	55	53.4	1.415	0.234
**Have you or any person you know been isolated due to NTDs? (n=95 at pretest, 103 at posttest)**						
Yes	44	46.3	36	35.0		
No	51	53.7	67	65.0	2.651	0.104
**Have your children or those of anyone you know been sent out of school because of NTDs? (n=95 at pretest, 103 at posttest)**						
Yes	34	35.8	34	33.0		
No	61	64.2	69	67.0	0.169	0.681
**Have you or anyone you know been prevented from entering a car because of NTDs?**						
Yes	38	40.0	40	38.8		
No	57	60.0	63	61.2	0.028	0.867
**Would you sit in the same car with anyone with any NTD?**						
Yes	99	27.9	152	42.0		
No	161	45.4	107	29.6	22.07	0.000
**Would you allow your children to attend school where there is a known NTD case?**						
Yes	80	22.5	156	43.1		
No	180	50.7	103	28.5	45.42	0.000
**Would you employ anyone with NTD?**						

Yes	53	14.9	117	32.3		
No	207	58.3	142	39.2	36.198	0.000
**Would you continue to be friends if any of your friends suddenly develop NTDs?**						
Yes	108	30.4	156	43.1		
No	152	42.8	103	28.5	18.141	0.000
**Would you patronize a seller with any of the NTDs?**						
Yes	79	22.3	131	36.2		
No	181	60.0	128	35.4	21.965	0.000
**Would you render assistance to anyone with obvious NTD?**						
Yes	114	32.1	153	42.3		
No	146	41.1	106	29.3	12.044	0.001

This significant reduction in stigmatization associated with NTDs is shown in the KII where some of the key informants have this to say regarding change in attitude *''My community can now understand that these are diseases and not as a result witchcraft, and therefore welcome everyone in the society'' (TL).*

The discrimination and fear of contracting the disease has reduced to a resonable extent" (YL2)

"People with NTDs can associate freely in the society with hope of getting cure to the disease" (WL1)

Many in the community now believe that these diseases were not as a result of curse from gods (RL2)

Respondents' perception about the Public Artwork

Two additional questions were added to the follow-up questionnaire, with the intention of gauging the people's perception about the public are initiative. Table 8 shows that 95.3% of the follow-up participants had encountered the Public Art murals in the community, and 98.3% of them want the Artwork to be maintained in the community.

Table 8: Respondents' perception about the Public Artwork

**Have you seen the NTDs Public Art in this community?**		
**Responses**	Frequency	Percent
Yes	345	95.3
No	17	4.7
**Total**	**362**	**100**

Do you want the Artwork to be maintained in this community? n= 345		
Yes	339	98.3
I don't care	6	1.7
**Total**	**362**	**100**

Discussion

The use of public art as a health intervention represents an innovative approach for improving knowledge, attitudes, and practices regarding NTDs, particularly in local communities where misconceptions and stigma hinder effective disease control. This study sought to assess the influence of community public art in enhancing knowledge, reshaping attitudes, encouraging preventive practices, and reducing social stigmatization surrounding NTDs in Okwelle Community, Imo State, Nigeria. The findings provide strong evidence of its effectiveness in achieving these goals.

Baseline knowledge of NTDs in the study area was generally poor, with only a small proportion of respondents having prior awareness of the diseases including onchocerciasis, Buruli ulcer and Lymphatic filariasis. This aligns with findings from Jaiyeola et al.(13) in an endemic town in Oyo State, Nigeria, where most participants had poor knowledge of lymphatic filariasis. Similarly, Fitzpatrick et al. (14) reported widespread gaps in NTD awareness, reinforcing the need for innovative health education strategies. Conversely, a study by Olamiju et al. (11) in Nigeria reported higher awareness levels, suggesting that knowledge disparities may be influenced by sociocultural factors and previous exposure to health campaigns.

The intervention led to significant improvements in knowledge, with more respondents correctly identifying NTD manifestations and understanding their risk. More importantly, awareness of Onchocerciasis rose from 28.5% to 39%, while knowledge of Buruli ulcer and lymphatic filariasis also increased significantly. Improved recognition of disease symptoms and transmission patterns further supports the argument that community-based interventions play a crucial role in bridging knowledge gaps

[15]. The success of this public art intervention suggests that visual and participatory education methods may be more effective than conventional health education approaches in rural communities.

Prior to the intervention, attitudes toward NTDs were characterized by misconceptions and deeply ingrained stigma. Many participants held beliefs that NTDs were hereditary, a result of witchcraft, or divine punishment. Similar attitudes have been documented in previous studies, where supernatural explanations were common barriers to seeking medical treatment [16]. However, after exposure to the public art, there was a substantial shift in perceptions. For instance, the proportion of respondents who disagreed that NTDs were a curse from God increased significantly. This aligns with findings from Alonso & Alvar, (2010) [17], which highlight that stigma and discrimination against individuals with NTDs often stem from fear and misinformation. Reducing stigma is essential for increasing healthcare-seeking behaviors and promoting social inclusion. The success of this intervention demonstrates that using culturally relevant and visually engaging methods can foster empathy, dispel myths, and encourage positive attitudinal changes in affected communities.

Baseline findings revealed poor health-seeking behaviors, with only 26% seeking medical treatment for NTD-related symptoms. This low engagement in healthcare services is consistent with prior studies that have linked poor knowledge to inadequate preventive behaviors [15]. However, post-intervention findings showed a significant increase in preventive practices, with participation in community health programs rising from 47.8% to 71.3% and active sharing of NTD-related information increasing from 37.8% to 68.0%. The improvement in preventive measures, such as personal hygiene and protective clothing use, supports earlier research indicating that targeted educational campaigns are crucial for behavior change [18]. Moreover, the increase in respondents who believed that treatment drugs should be prescribed by healthcare professionals (from 73.5% to 85.4%) suggests a growing trust in formal medical interventions, a crucial step toward effective disease control.

One of the most notable findings was the reduction in stigmatization. While stigma-related barriers to healthcare access have been widely documented [16], this study provides empirical evidence that public art interventions can actively counteract these social barriers. Willingness to associate with individuals affected by NTDs improved across multiple indicators, including willingness to sit in the same vehicle (27.9% to 42%) and employ affected individuals (14.9% to 32.3%). These findings highlight the potential for community-driven interventions to challenge stereotypes and promote inclusivity.

Additionally, qualitative insights from key informants reinforced this shift in perception. Participants expressed greater acceptance of individuals with NTDs, recognizing that these diseases were neither supernatural nor untreatable. This aligns with previous findings that stigma reduction is a critical component of effective disease management interventions [18]. The ability of public art to evoke emotional responses and facilitate dialogue suggests its viability as a scalable tool for addressing stigma across different NTD-endemic regions.

The overwhelming acceptance of the public art initiative (95.3% of respondents reported encountering the murals, and 98.3% wanted them to remain) underscores its relevance as a community-driven health education strategy. Given its success, integrating public art into mainstream health communication strategies could enhance the reach and impact of NTD awareness campaigns.

The findings from this study have important implications for other NTD-endemic regions. Public art interventions, when designed with community participation, can be adapted to various socio-cultural contexts to enhance public health education. This aligns with global efforts to integrate creative, community-driven strategies into disease control programs. Furthermore, increasing NTD knowledge and reducing stigma can lead to higher rates of early diagnosis and treatment adherence, ultimately supporting disease elimination goals.

Strengths and Limitations

A key strength of this study is its mixed-methods approach, combining quantitative surveys with qualitative insights from key informant interviews and focus group discussions. This allowed for a comprehensive evaluation of the intervention's impact. Additionally, the use of community-based participatory research ensured strong local engagement, which may have contributed to the effectiveness of the intervention.

However, some limitations should be acknowledged. The study was conducted within a single community, which may limit the generalizability of the findings to broader populations. Additionally, while short-term improvements in knowledge and attitudes were observed, long-term retention and behavioral changes were not assessed. Future studies should incorporate follow-up assessments to determine whether these changes are sustained over time. Potential biases in self-reported data should also be considered, as social desirability may have influenced responses.

Impact on Existing Knowledge

This study builds on previous research demonstrating the role of culturally relevant interventions in health education. Prior studies have highlighted the effectiveness of visual storytelling and participatory methods in increasing health literacy [19]. However, few studies have specifically evaluated public art as a tool for NTD awareness and stigma reduction. The significant improvements observed in this study suggest that public art could complement existing health education strategies, particularly in low-literacy settings where conventional approaches may be less effective.

Conclusion and Future Directions

The findings from this study suggest that public art interventions can serve as a powerful, scalable strategy for enhancing NTD awareness, improving attitudes, and reducing stigma. Compared to traditional health education methods, public art provides an engaging, culturally relevant, and visually impactful approach

to disease control. Future research should explore the long-term sustainability of such interventions, assess their replicability in different socio-cultural contexts, and investigate their impact on healthcare-seeking behaviors over time. Strengthening collaborations between public health professionals, artists, and policymakers could further optimize this approach and contribute to global NTD elimination efforts.

Acknowledgments

We extend our sincere gratitude to the Community Health Extension Workers (CHEWs) and Community Health Officers (CHOs) at the Okwelle Basic Health Center for their invaluable support during this study. We also appreciate the traditional leader, religious leaders, and other community stakeholders who actively participated in the Focus Group Discussions (FGDs) and Key Informant Interviews (KIIs).

Special thanks to the Department of Public Health, Federal University of Technology, for reviewing the study protocol and providing ethical clearance. We acknowledge the dedicated efforts of our research assistants whose contributions were instrumental in data collection and community engagement. Finally, we deeply appreciate the study participants for their willingness to share their experiences and insights, making this research possible.

References

1. WHO. Health Topics. 2024 [cited 2024 Jan 4]. Neglected Tropical Diseases. Available from: https://www.who.int/health-topics/neglected-tropical-diseases#tab=tab_2
2. Hotez PJ. Forgotten people, forgotten diseases: the neglected tropical diseases and their impact on global health and development. John Wiley & Sons; 2021.
3. Wilsher E. The impact of Neglected Tropical Diseases, and their associated stigma, on people’s basic capabilities. Durham University; 2011.
4. Weiss MG. Stigma and the social burden of neglected tropical diseases. PLoS Negl Trop Dis. 2008;2(5):e237.
5. Hou SI. Health education: theoretical concepts, effective strategies and core competencies. Health Promot Pract. 2014;15(5):619–21.

6. Organization WH. Health education: theoretical concepts, effective strategies and core competencies. 2012. 2017.

7. Anoko JN, Barry BR, Boiro H, Diallo B, Diallo AB, Belizaire MR, et al. Community engagement for successful COVID-19 pandemic response: 10 lessons from Ebola outbreak responses in Africa. BMJ Glob Health. 2020;4(Suppl 7):e003121.

8. Fancourt D, Finn S. What is the evidence on the role of the arts in improving health and well-being? A scoping review. World Health Organization. Regional Office for Europe; 2019.

9. Clift S. Creative arts as a public health resource: moving from practice-based research to evidence-based practice. Perspect Public Health. 2012;132(3):120–7.

10. Kish L. Multipurpose sample designs. Leslie Kish: Selected Papers. 2003;330:253.

11. Olamiju OJ, Olamiju FO, Adeniran AA, Mba IC, Ukwunna CC, Okoronkwo C, et al. Public awareness and knowledge of neglected tropical diseases (NTDs) control activities in Abuja, Nigeria. PLoS Negl Trop Dis. 2014;8(9):e3209.

12. IBM Corporation. Statistical Package for Social Science (SPSS) [Internet]. Chicago, IL; 2021. Available from: www.ibm.com/products/spss-statistics

13. Jaiyeola TM, Udoh EE, Adebambo AB. Knowledge, attitude, and practice towards lymphatic filariasis among inhabitants of an endemic town in Oyo State, Nigeria. medRxiv. 2022;2022–5.

14. Fitzpatrick C, Nwankwo U, Lenk E, de Vlas SJ, Bundy DAP. An investment case for ending neglected tropical diseases. Major Infectious Diseases 3rd edition. 2017;

15. Haldane V, Chuah FLH, Srivastava A, Singh SR, Koh GCH, Seng CK, et al. Community participation in health services development, implementation, and evaluation: A systematic review of empowerment, health, community, and process outcomes. PLoS One. 2019;14(5):e0216112.

16. Hofstraat K, van Brakel WH. Social stigma towards neglected tropical diseases: a systematic review. Int Health. 2016;8(suppl_1):i53–70.

17. Alonso LM, Alvar J. Stigmatizing neglected tropical diseases: a systematic review. Social Medicine. 2010;5(4):218–27.

18. Lenka D, Mahapatra A. Role of Reconstructive Surgery (RCS) in Improving the Quality of Life of Leprosy Afflicted Persons. Indian J Lepr. 2016;88(1):7–12.

19. Galmarini E, Marciano L, Schulz PJ. The effectiveness of visual-based interventions on health literacy in health care: a systematic review and meta-analysis. BMC Health Serv Res. 2024;24(1):718.

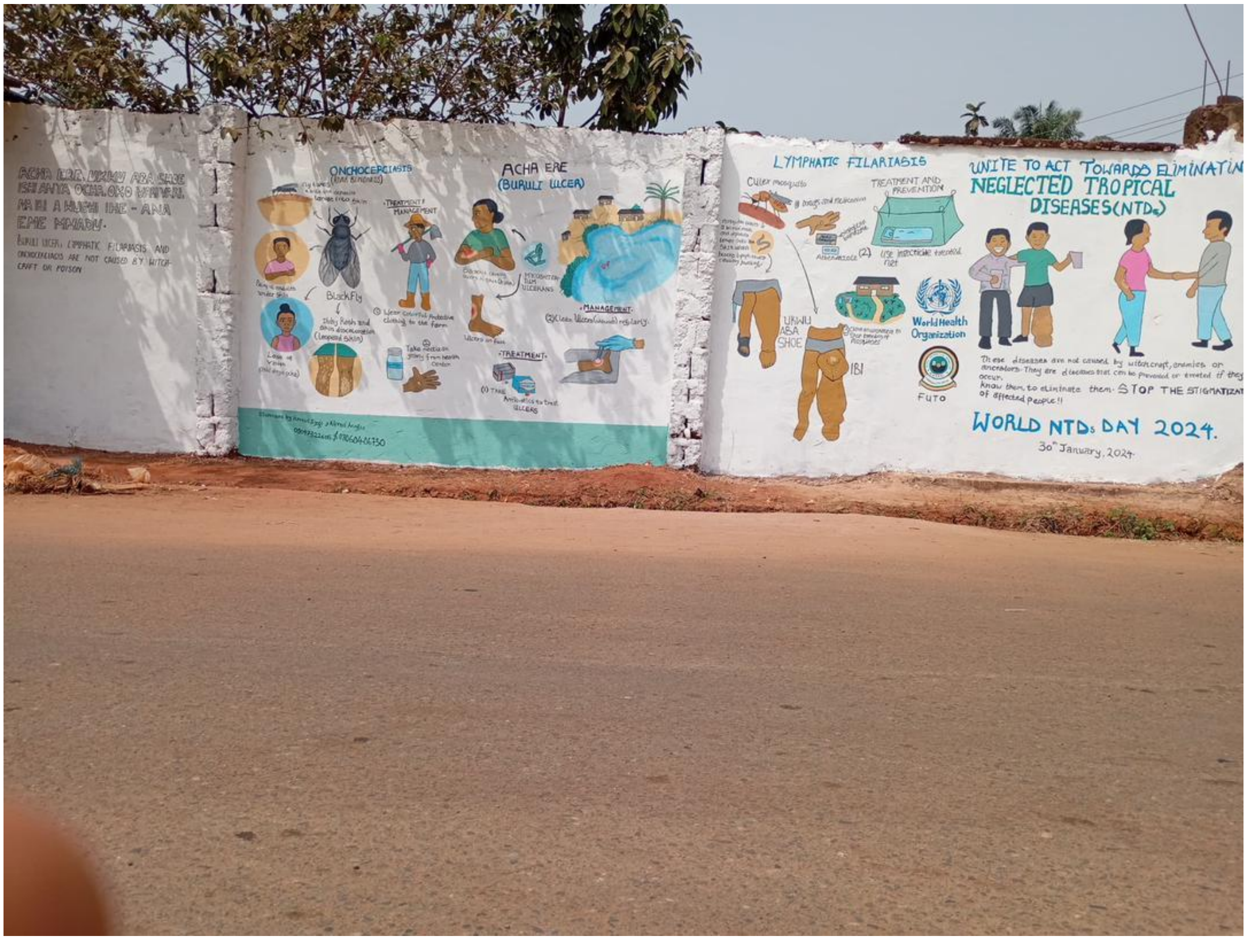

Plate 1: **The view of public artwork displayed in a conspicuous location in the study community**
