# Supplementary material for: Assessment of the effectiveness of public art in improving knowledge, attitude, practices and mitigation of stigmatization regarding neglected tropical diseases in South Eastern, Nigeria
Source: PLoS Negl Trop Dis. 2025 Jul 1;19(7):e0013266. doi: 10.1371/journal.pntd.0013266 (PMC12212551; doi:10.1371/journal.pntd.0013266)
Supplement: Attachment — Submitted filename: re-comments NTD article.docx [file pntd.0013266.s006.docx]

Most comments from the first review have been corrected, but a few others were not corrected and a few additional comments

Abstract states that 361 respondents were used, the main body says 362 respondents, please correct

The narrative in the results have been slightly improved but it can be further enhanced to improve the flow

In Table 1, age can appear as mean age, then the mean ± SD can be presented for both pre-test and post-test

Line 400, and across the entire quantitative result section, there is no need to put the frequency of respondents in brackets; simply use the percentage, the n is already displayed on the tables

Line 406, uncertainty about what? Please complete the sentence

Line 416, add **from** person to person

In tables 2-6, questions are still on the tables, since data have been collected, questions should appear as variables in the result section. Correct for tables 2-6

e.g:

- Ever heard of Onchocerciasis
- Identifying oncho manifestation from the photo album
- Individuals mostly affected by NTDs
- Problems associated with NTDs
- Ways oncho is contracted
- Ways to prevent oncho
- Protective measures against oncho
- Treatment choices

Apply to tables 3-4

Line 483, and across the entire table 5 narrative, there is no need to keep putting disagree and strongly disagree in bracket if both variables have been collapsed, same with agree and strongly agree. The result section should be summarized, you can simply use the term agree or disagree

Table 6, variables too long, shorten, and don’t repeat questions e.g:

- Ever sought medical treatment for NTD-related symptoms in the past year
- Frequency of engaging in NTD prevention practices
- Participation in NTD- related community health programs
- Active sharing of NTD related info
- NTD medication should be prescribed by a doctor

There’s no need to put total after each variable, put instead on the heading n = 362

Table 7: (n=95 at pretest, 103 at posttest) should come under the respective heading once and not after each variable

Line 550: names of authors shouldn’t be mentioned at all within text, stick to journal’s recommendation, numberings alone should appear within text

Table 8, Seen the NTDs Public Art in the community can appear as a variable and not heading, same with the second heading n = 362 should come under the variable.

There is no need for total after each variable once n= 362 is added. NR can be used to account for the remainder if there was actually no response

Lines 599, 601, 619, remove author names with text
